# Molecular Dynamics Investigation of the Influence of Voids on the Impact Mechanical Behavior of NiTi Shape-Memory Alloy

**DOI:** 10.3390/ma14144020

**Published:** 2021-07-18

**Authors:** Zhenwei Wu, Xiang Chen, Tao Fu, Hengwei Zheng, Yang Zhao

**Affiliations:** 1 School of Advanced Manufacturing Engineering, Chongqing University of Posts and Telecommunications, Chongqing 400065, China; 2018214482@stu.cqupt.edu.cn (Z.W.); zhaoyang@cqupt.edu.cn (Y.Z.); 2 School of Aeronautics and Astronautics, Chongqing University, Chongqing 400044, China; futaocqu@163.com; 3 State Key Laboratory of Mechanical Structure Strength and Vibration, Xi’an Jiaotong University, Xi’an 710049, China; 4 School of Civil Engineering and Architecture, Chongqing University of Science & Technology, Chongqing 401331, China

**Keywords:** shape-memory alloy, NiTi alloy, voids, molecular dynamics simulation, shock behavior, martensitic transformation

## Abstract

To date, research on the physical and mechanical behavior of nickel-titanium shape-memory alloy (NiTi SMA) has focused on the macroscopic physical properties, equation of state, strength constitution, phase transition induced by temperature and stress under static load, etc. The behavior of a NiTi SMA under high-strain-rate impact and the influence of voids have not been reported. In this present work, the behavior evolution of (100) single-crystal NiTi SMA and the influencing characteristics of voids under a shock wave of 1.2 km/s are studied by large-scale molecular dynamics calculation. The results show that only a small amount of B2 austenite is transformed into B19’ martensite when the NiTi sample does not pass through the void during impact compression, whereas when the shock wave passes through the hole, a large amount of martensite phase transformation and plastic deformation is induced around the hole; the existence of phase transformation and phase-transformation-induced plastic deformation greatly consumes the energy of the shock wave, thus making the width of the wave front in the subsequent propagation process wider and the peak of the foremost wave peak reduced. In addition, the existence of holes disrupts the orderly propagation of shock waves, changes the shock wave front from a plane to a concave surface, and reduces the propagation speed of shock waves. The calculation results show that the presence of pores in a porous NiTi SMA leads to significant martensitic phase transformation and plastic deformation induced by phase transformation, which has a significant buffering effect on shock waves. The results of this study provide great guidance for expanding the application of NiTi SMA in the field of shock.

## 1. Introduction

Shape-memory alloys (SMAs) are alloy materials that recover their original shape before deformation by heating the material to a critical temperature and, at the same time, can have large recoverable elastic strain under unloading. The former property is called the shape-memory effect (SME), and the latter is called super-elasticity (SE) [1,2,3]. Among shape-memory alloys, the NiTi SMA is widely used in machinery, microelectromechanical systems, aerospace, medical devices, and biomedicine because of its many excellent properties, such as its wider shape-memory effect, super-elasticity effect, good biocompatibility, wear resistance, and corrosion resistance [4,5,6]. The porous NiTi shape-memory alloy is a highly dampened material for vibration damping, which has stronger damping properties than general metallic materials due to its large number of microporous structures and large number of grain boundaries in the tissue. It also has a greater prospect of application in vibration damping devices and is potentially valuable in reducing the effects of shocks and explosions [7,8,9].

In recent decades, domestic and foreign scholars have reported the macroscopic physical properties, thermal-mechanical properties, and multidimensional intrinsic structure models of NiTi SMA. In recent years, with the development of macro- and microscopic numerical simulation techniques, reports on the microstructural evolution related to shape-memory effect and super-elasticity have gradually increased [10,11,12]. However, most of these reports focus on the effects of static loading and temperature changes, with only a few reports on the phase transformation, twinning, and detwinning of NiTi SMAs under high strain rate impact loading. Yu et al. [13] studied the influence of heat treatment on the mechanical properties of austenitic NiTi alloys during impact loading, reporting that Ni4Ti3 precipitates can improve the pseudo-elasticity of NiTi SMAs during impact loading. Wang et al. [14] investigated the thermodynamic behavior of NiTi SMA sheets subjected to low-velocity impact loading by means of experiments and explicit finite element (FE) analysis, an analysis approach that captured the prime thermodynamic features of the SMAs against low-velocity impact loads such as superelastic deformation, stress-induced martensite phase transformation, and temperature variation. Gur et al. [15] compared the simulation results for nanoporous NiTi with various porosity configurations with non-porous NiTi, finding that the martensite phase fraction and transformation temperature increase significantly with the increase in porosity, the stress-strain response changes significantly with the change in porosity, and the residual strain and hysteretic energy dissipation capacity increase significantly with the increase in porosity.

Molecular dynamics (MD)-based simulation methods have been studied for more than half a century, and their applications are becoming more and more widespread due to the low research cost and high reproducibility. From the time scale, the classical equations of motion in molecular dynamics simulations can be solved numerically for each atom in the system as opposed to actual experiments [16], and each time step of the overall system can be recorded to obtain the microscopic details of the model at each time step. Therefore, molecular dynamics simulation methods are well suited for the study of shock loading behavior [17]. In molecular dynamics simulations, the interatomic interaction potential is the key to the simulations. For the Ni-Ti binary potential function, Lai et al. [18] applied the embedded Finnis–Sinclair (EAM-FS) atomic potential for the first time to describe the interatomic interactions of NiTi alloys. After that, Zhong et al. [19] improved the potential function established by Lai et al. to study the phase transformation and mechanical behavior of shape-memory alloys under compressive loading and unloading. Based on this potential function, Yazdandoost et al. [20] investigated the energy dissipation in NiTi alloys using atomic-scale simulations, and found that the energy dissipation in NiTi alloys was caused by both phase transformation and plastic deformation, and the stress waves were significantly and effectively attenuated in NiTi alloys by phase transformation, while plastic deformation did not contribute much to the energy dissipation. In addition, Yazdandoost and Mirzaeifar [21] used this potential function to study the energy dissipation and phase transformation of single-crystal and polycrystalline NiTi alloys under impact loading at the atomic scale using molecular dynamics simulations, as well as the effects of lattice orientation, grain boundaries, and grain size on the propagation of shock waves and shock-wave-induced phase transformation propagation. Wang et al. [22] used molecular kinetic simulations to reveal the evolution of the martensitic phase transition, martensitic inverse phase transition, and twinning behavior of isoatomic NiTi SMAs under the effect of shock compression.

At present, most of the molecular dynamics simulations of NiTi SMAs focus on the temperature and stress-induced phase transformation under static loading, and there are few studies involving the phase transformation and mechanical behavior under high strain rate impact loading, while studies of the effect of pores on NiTi SMAs under high strain rate impact are rarely reported. Therefore, in this paper, the impact response of NiTi SMAs is investigated by means of molecular dynamics simulation. The study focuses on the atomic structure characteristics, energy distribution characteristics, and evolution of holes and dislocations at different moments of the impact compression stage with and without holes, and it is supported by the propagation curves of the atomic average temperature, stress, velocity, and other relevant physical quantities along with the above study. The phase transformation and mechanical behavior of the NiTi SMA in the impact compression stage under the action of high-strain-rate shock waves and the effect of holes on the phase transformation and mechanical properties of the NiTi SMA in the compression stage under the action of shock waves are further revealed, which are important for the study of the shape-memory effect and super-elasticity of NiTi SMAs at a large strain rate.

## 2. Materials and Methods

### 2.1. Computational Modeling

**Figure 1:**
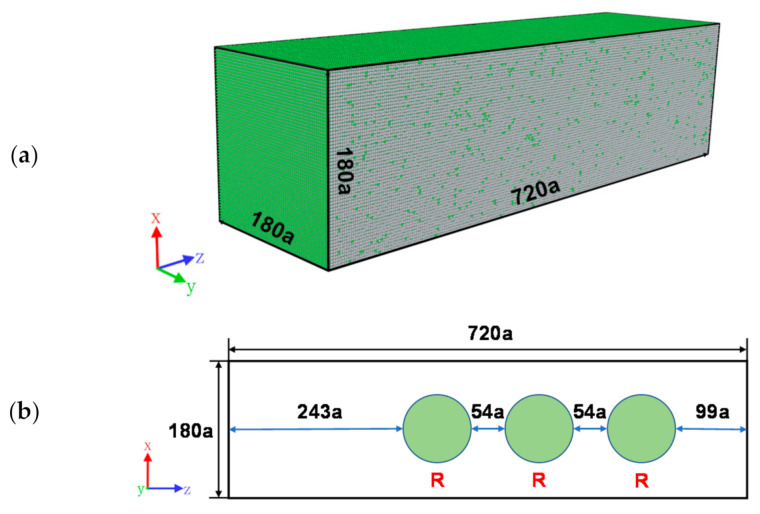
Molecular dynamics model with B2 phase as initial structure: (**a**) non-porous sample; (**b**) porous sample (R = 45 a).

In this study, the initial structures of the molecular dynamics models established for both non-porous and porous samples are B2 phase (body-centered cubic structure), and the specific parameters of their model dimensions are shown in Table 1. The distribution of the pores is shown in Figure 1b, where the radius of the pores R = 45 a. In addition, the three axes x, y, and z correspond to the [100], [010], and [001] directions, respectively.

### 2.2. Interatomic Potential

Since the interaction potential between atoms determines the interaction performance and fundamental properties of the material, the correct choice of interaction potential between atoms is the key to molecular dynamics simulations. In this paper, the potential function used for molecular dynamics simulations is based on the EAM potential function of the embedded atom approach developed by Lai and Liu et al. [18] and later improved by Zhong. Its expression is given by the following:

(1) $$E=\sum_{i}\left\{\sum_{j\neq i}A_{\alpha \beta }\exp\left[-p_{\alpha \beta }\left(\frac{r_{ij}}{d_{\alpha \beta }}-1\right)\right]-\sqrt{\sum_{j\neq i}F(r_{ij})}\right\}$$

where

(2) $$F\left(r_{ij}\right)=\xi_{\alpha \beta }^{2}\exp[-2q_{\alpha \beta }\left(\frac{r_{ij}}{d_{\alpha \beta }}-1\right)]$$

where $r_{ij}$ is the distance between different atoms *i* and *j*, and *α* and *β* represent different atom types. The first term in Equation (1) represents a pair interaction and the second term represents a many-body effect. The potential function Equation (2) was further improved by Zhong et al. [19,23] as Equation (3).

(3) $$F\left(r_{ij}\right)=\{\begin{array}{l} \xi_{\alpha \beta }^{2}\exp[-2q_{\alpha \beta }\left(\frac{r_{ij}}{d_{\alpha \beta }}-1\right)],\;r_{ij}\leq r_{1}\\ c_{3,\alpha \beta }{\left(r_{ij}-r_{1}\right)}^{3}+c_{2,\alpha \beta }{\left(r_{ij}-r_{1}\right)}^{2}+c_{1,\alpha \beta }(r_{ij}-r_{1})+c_{0,\alpha \beta },\;r_{1}<r_{ij}\leq r_{c} \end{array}$$

The improved potential function not only avoids the elimination of discontinuities in the derivatives of the potential function, but also improves the prediction of the fundamental properties of the material, including the lattice constants and cohesion energies of various phases. The potential function was fitted to the properties of phase B2 at 0 K according to the first-nature principal calculation, and the cut-off radius was determined to be 4.2 Å. The parameters of the optimized potential function are shown in Table 2.

### 2.3. Simulation Software

The used molecular dynamics software (large-scale atomic/molecular massively parallel simulator, LAMMPS, 1 Sep 2017 version) [24] is a general multifunctional molecular dynamics simulation software developed by Sandia National Laboratories, Albuquerque, NM, USA, based on the C++ language, which is open-source, free, and highly scalable; it can simulate a variety of model systems, and also supports efficient parallel computing, and its simulation system can reach hundreds of millions of particles at present.

Here, LAMMPS was used for the impact compression calculation of NiTi shape-memory alloy. The molecular dynamics simulation results were analyzed with the help of the scientific visualization and analysis software OVITO (Open Visualization Tool, 3.4.1 version, Darmstadt, Germany) [25]. The polyhedral template comparison method (PTM), color coding of atomic kinetic energy, and the dislocation extraction algorithm (DXA) were adopted to show more clearly the changes in atomic structure characteristics, energy distribution characteristics, changes in defects such as dislocations and stacking layer dislocations, and changes in related physical quantities of the samples during the impact process for the impact of NiTi shape-memory alloy without holes. Furthermore, comparative analysis of non-porous NiTi SMA samples and porous NiTi SMA samples was carried out. Among the methods, the PTM was not originally designed to distinguish between B2, B19, and B19’ phases, but it possesses this capability as confirmed by Chen et al. [10,11,12], Ko et al. [26], and Wang et al. [27]. Because the PTM cannot identify the B19’ phase structure, the body-centered cubic (BCC) structure is the B2 phase, the face-centered cubic (FCC) structure is the B19 phase, and the dense-row hexagonal (HCP) structure is the B19’ phase by default.

### 2.4. Shock Loading Conditions

**Figure 2:**
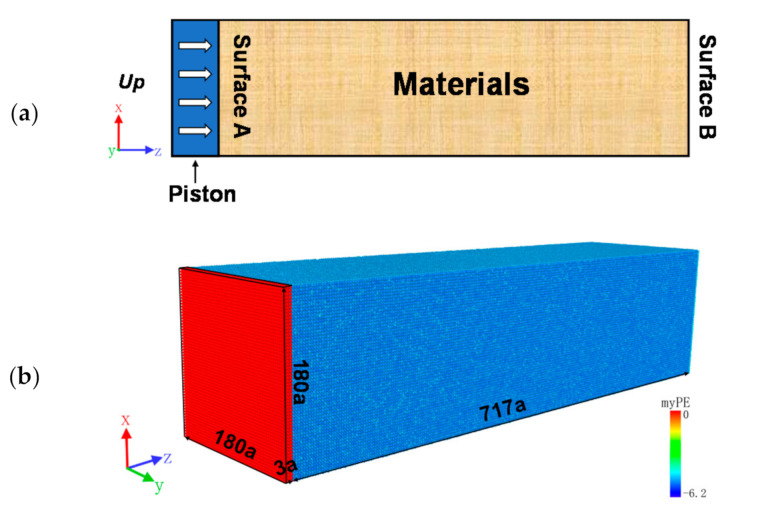
Multidimensional simulation model of piston impact load: (**a**) schematic diagram of piston impact method; (**b**) demonstration of molecular dynamics model in OVITO.

Before the impact loading, the model was isothermally and isobarically (NPT system) relaxed at 500 K and zero pressure in order to relieve the stresses inside the model and bring the sample to thermodynamic equilibrium. After the relaxation was completed, a shock wave was generated by the “piston impact method” [28,29]. The schematic diagram of piston impact is shown in Figure 2a, where the piston moves with velocity +Up, pushing the relatively stationary material and generating shock waves in it. This approach uses periodic boundary conditions in the x and y directions of the model, while using non-periodic boundary conditions in the z direction of the impact. Based on the molecular dynamics model shown in Figure 2b, six layers of atoms with a total thickness close to 3 were set up as shock pistons, and the atomic potential PE was colored using OVITO software to distinguish between the pistons and the target sample, where the red part on the left side represents the pistons and the other part represents the material.

When the shock wave reaches the free surface, it will lead to a reflection of the compressional shock wave depending on the impedance rules for waves passing through different environments. Therefore, the compression shock wave becomes a stretching shock wave after reaching the free boundary. The shock wave propagates from surface A to surface B for forward compression in the first stage, while the shock wave returns from surface B to surface A for reverse stretching in the second stage. Subsequently, the shock wave propagates again from surface A to surface B in the third stage, and again, the shock wave returns from surface B to surface A in the fourth stage. The first and second stages can be considered a complete cycle of the shock wave. Similarly, the third and fourth stages can be considered another complete cyclic process of the shock wave. In fact, these four stages keep alternating during the propagation of the shock wave, and this paper focuses on the representative shock compression stage.

The relaxation process before shock loading was 40,000 time steps, of which 1 time step was 0.001 picosecond (ps). The shock loading process was 30,000 time steps, the shock wave direction was along the [100] crystal direction, and the piston generated a driving velocity of magnitude 1.2 km/s.

## 3. Results and Discussion

### 3.1. Atomic Structure Characteristics of the Shock Compression Phase

**Figure 3:**
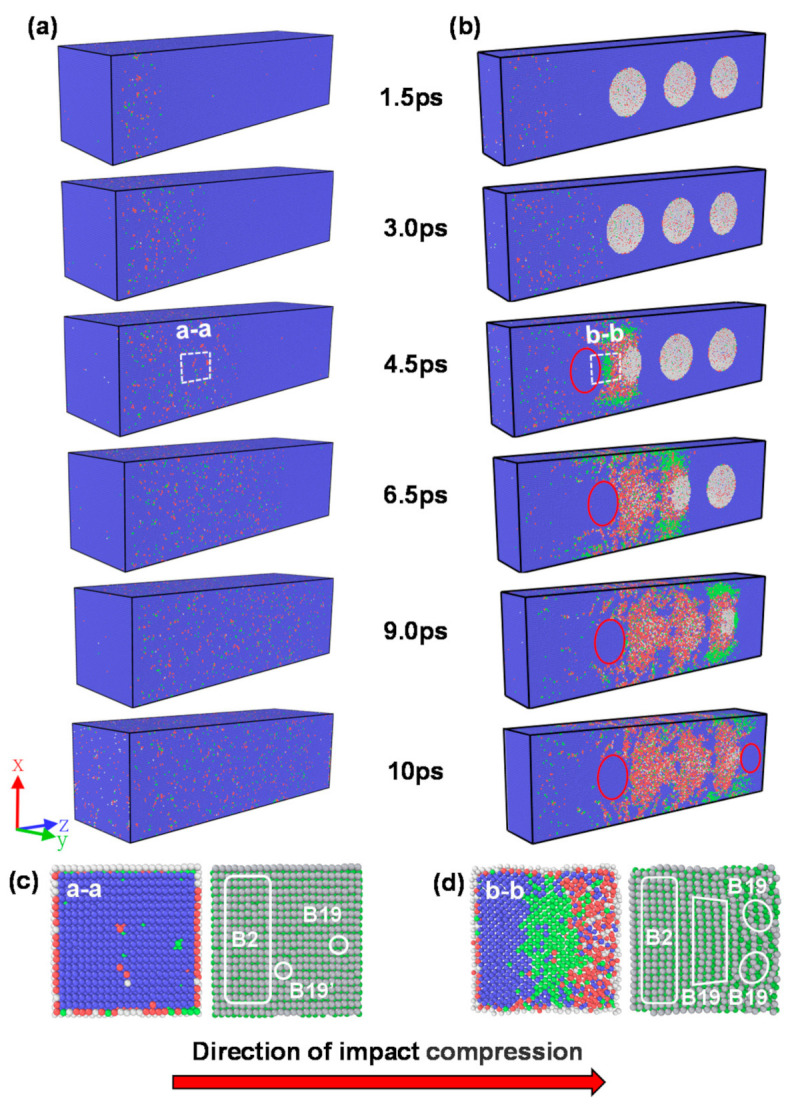
Atomic structure characteristics of non-porous samples and porous samples during impact compression: (**a**) non-porous samples; (**b**) porous sample profile (blue, red, green, and white atoms represent B2 phase, B19’ phase, B19 phase, and others, respectively); (**c**) cross-sectional view of the white dashed box a-a on the porous sample at 4.5 ps in Figure 3a (the left side is the structure diagram of PTM analysis, and the right side is the original structure diagram); (**d**) cross-sectional view of the white dashed box b-b on the non-porous sample at 4.5 ps in Figure 3b (the left side is the structure diagram of PTM analysis, and the right side is the original structure diagram).

Figure 3 shows the results of the atomic structure evolution of the NiTi SMA samples with/without holes at different moments during the first stage of shock wave propagation. Figure 3a shows that the shock wave propagates uniformly from left to right within the non-porous sample with time, and the B19 phase dominates at the front part of the shock wave, indicating that the B2 phase is transformed into the B19 phase first and then into the B19’ phase under the action of the shock wave. Additionally, it can be seen from Figure 3b that when the shock wave touches the hole, the hole shape changes and is squeezed along the hole direction, while a large amount of phase change and plastic deformation is generated around the hole, and the front of the phase change is still dominated by the B19 phase. When the shock wave passes through the hole, there is no propagation medium at the hole, so the shock wave needs to propagate forward along the edge of the hole, and the distance traveled by the shock wave increases. Naturally, the speed of shock wave propagation decreases, and the speed of the shock wave gradually slows down from the edge of the hole to the center of the hole. Al-Qananwah et al. [30] indicated that a porous material protective layer can effectively reduce the peak value of a stress wave and impact kinetic energy by about 30% by performing molecular dynamics simulations. Phase transitions and plasticity zones are formed around the original hole, and the structure is identified as a disordered mixed-phase structure; under the influence of the hole on the shock wave, the phase change and plasticity zones within the sample and plasticity regions in the sample increase significantly. Figure 3c,d shows the details of the phase transitions of the non-porous/porous samples at the moments of 4.5 ps. It can be seen that the phase transitions of the porous samples are concentrated around the holes. This coincides with the findings of Davila et al. [31], who studied the microstructural changes in ductile porous metals during high strain rate loading by atomic simulations.

**Figure 4:**
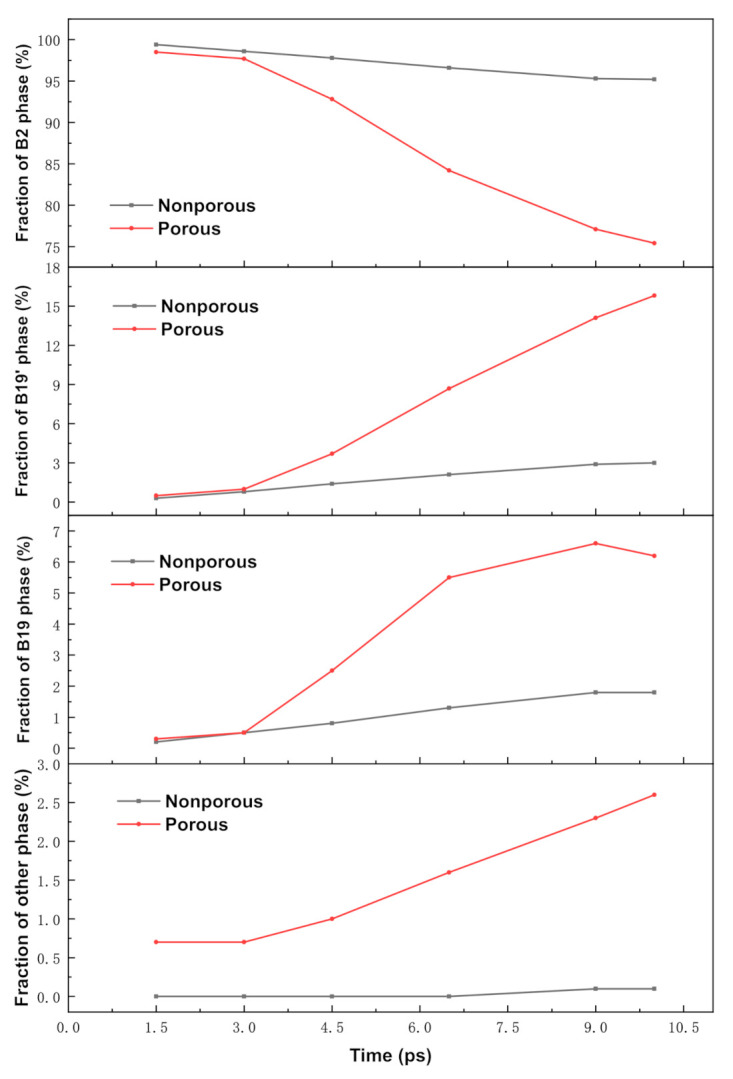
Microstructure components of non-porous and porous samples after forward compression loading phase change wave (obtained by PTM analysis).

Figure 4 shows the changes in the atomic structure of the region after the shock wave front for the samples without holes and with multiple holes. It can be seen from Figure 4 that the atomic structure of the non-porous sample changes less during the loading of the shock wave, and the B2 phase is always above 95%. Compared to the non-porous sample, the atomic structure change of the porous sample in the first phase is relatively large. During the time length from *t* = 1.5 ps to *t* = 10 ps, the porous sample decreases from the initial B2 phase structure with a fraction of 98.55% all the way down to 75.4%, the B19 phase fraction increases from 0.3% to 6.2%, and the B19’ phase changes more, from 0.5% to 15.8%. When the simulation time was 10 ps, the front end of the shock wave reached the surface B.

The mechanical behavior of NiTi shape-memory alloys is closely related to the phase transition behavior. Many researchers have demonstrated that stress-induced martensitic phase transformation of NiTi shape-memory alloys may occur during tensile, compressive, and shear loading [32,33,34]. Wang et al. [35,36] provided experimental evidence that NiTi shape-memory alloys undergo stress-induced martensitic transformation when subjected to laser impact strengthening, and its strain rate is about 10^7^ s^−1^. Their results suggest that the mechanism of stress-induced martensitic phase transformation at ultra-high strain rates is significantly different from that at low strain rates. In this paper, we corroborated the mechanism of stress-induced martensitic phase transformation at ultra-high strain [16], and revealed the effect of pores on the stress-induced martensitic phase transformation of NiTi shape-memory alloys. This has important implications for the study of stress-induced martensitic phase transformation in NiTi shape-memory alloys with non-porous and porous structures under impact loading conditions.

### 3.2. Energy Propagation Trajectory in the Shock Compression Phase

**Figure 5:**
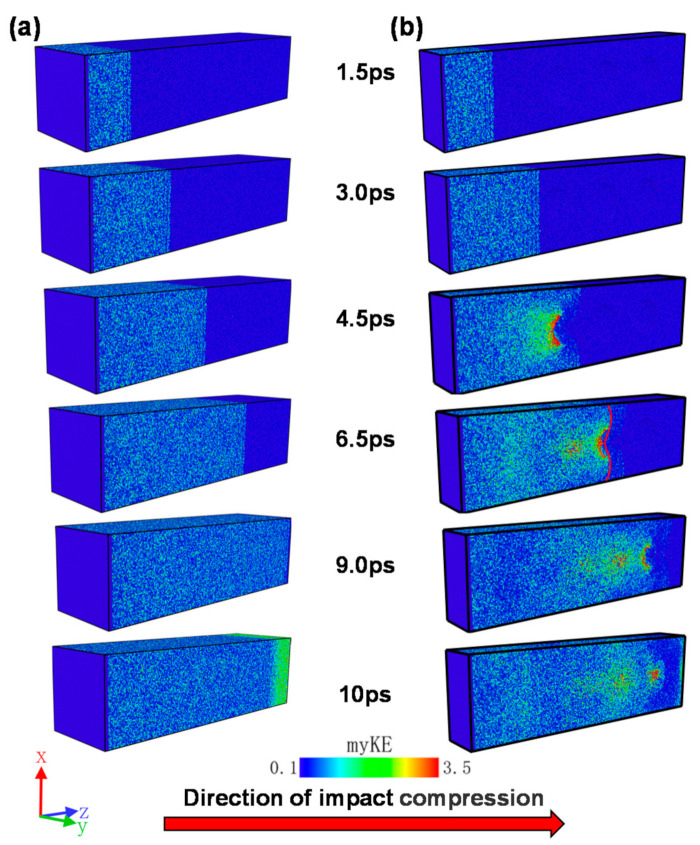
Energy propagation trajectories of non-porous samples and porous samples during impact compression: (**a**) non-porous samples; (**b**) porous samples.

The propagation trajectories (0–10 ps) of the shock wave during the shock compression phase for the non-porous and porous samples are shown in Figure 5. During the simulation, the kinetic energy of each atom was colored (color coding) using the OVITO software and the propagation of the shock wave was recorded, where myKE represents the kinetic energy of each atom.

Comparing Figure 5a,b, it can be observed that the kinetic energy of the atoms of the samples with and without holes propagates uniformly along the cross-section before the shock wave encounters the holes; at *t* = 4.5 to 6.5 ps in the figure, it is easy to see that when the shock wave encounters the holes, the energy of the atoms increases sharply in the vicinity of the holes, especially in the direction of the windward side of the holes along the propagation of the shock wave, and is much larger than the kinetic energy of the samples without holes, with a “V” shape filling the hole until it fills the hole area and continues to propagate backward. It is worth noting that the average kinetic energy of the atoms decreases significantly after the shock wave passes through the hole, as shown in the red curve corresponding to *t* = 6.5 ps in Figure 5b, where the kinetic energy of the atoms on the right side of the red line is significantly smaller than that of the non-porous sample, as well as the atoms on the left side of the red line, indicating that the shock wave has a significant energy loss after passing through the hole. In addition, comparing the non-porous and porous samples at the moment of *t* = 10 ps, it can be found that under the same sample size and impact velocity, the shock wave of the non-porous sample is already close to the surface B, yet the shock wave of the porous sample is significantly further away from the surface B. A more detailed comparison will be carried out in the following sections.

### 3.3. Pore and Dislocation Evolution in the Impact Compression Stage

**Figure 6:**
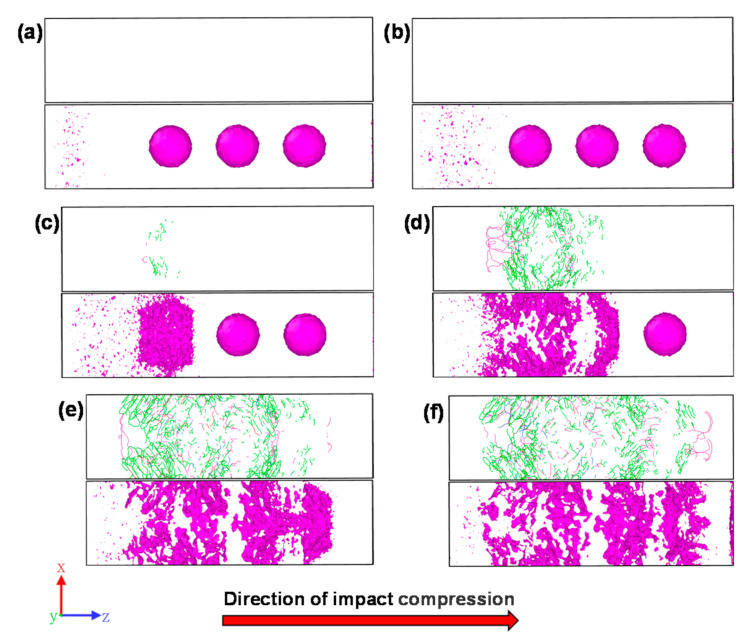
Evolution of voids and dislocations in porous samples during compression: (**a**) 1.5 ps; (**b**) 3.0 ps; (**c**) 4.5 ps; (**d**) 6.5 ps; (**e**) 9.0 ps; (**f**) 10 ps (pink is <100> dislocation, blue is <110> dislocation, green is ½ <111> dislocation, and red is other dislocation).

The results of the analysis of the evolution process of dislocations and internal defects in the porous sample at different moments of the shock compression stage are given in Figure 6. As seen in Figure 6a,b, no obvious dislocation lines were identified within the sample when the shock wave had not yet touched the holes, but obvious defects were generated in the sample area through which the shock wave propagated. It can be seen from Figure 6c that at the time when the shock wave passes through the first hole (*t* = 4.5 ps), obvious dislocations appear within the sample and the first hole partially collapses, forming a large number of defects; it can be seen from Figure 6d that at the time when the shock wave passes through the second hole (*t* = 6.5 ps), the first hole completely collapses, the defects expand to the perimeter of the hole, the second hole partially collapses, and the total amount of dislocations increases to about 20 times the amount at *t* = 4.5 ps. It is worth noting that, compared to Figure 3b, although the atomic region in front of the first hole still remains as a B2 structure at this time, a large number of <100> dislocations are generated inside; when comparing Figure 3b at *t* = 10 ps and Figure 6f, a similar phenomenon is observed on the right side of the third hole. In addition, comparing Figure 3, it can be seen that the dislocation region overlaps with the concentrated martensitic phase transformation region, indicating that the shock wave passing through the hole induces a large amount of martensitic phase transformation around the hole and produces phase-transformation-induced plastic deformation, and this process will consume a large amount of energy, which leads to the subsequent reduction in the energy of the shock wave front, as shown in Figure 5b. From Figure 6e, it can be seen that when the shock wave passes through the third hole (*t* = 9.0 ps), the first two holes collapse completely and the third hole collapses partially, and the total dislocation increases to about 33 times the amount at *t* = 4.5 ps; from Figure 6f, it can be seen that when the shock wave reaches surface B (*t* = 10 ps), the three holes collapse completely and the dislocation increases to about 35 times the amount at *t* = 4.5 ps.

### 3.4. Curve of Physical Quantity Change of Impact Compression Process

**Figure 7:**
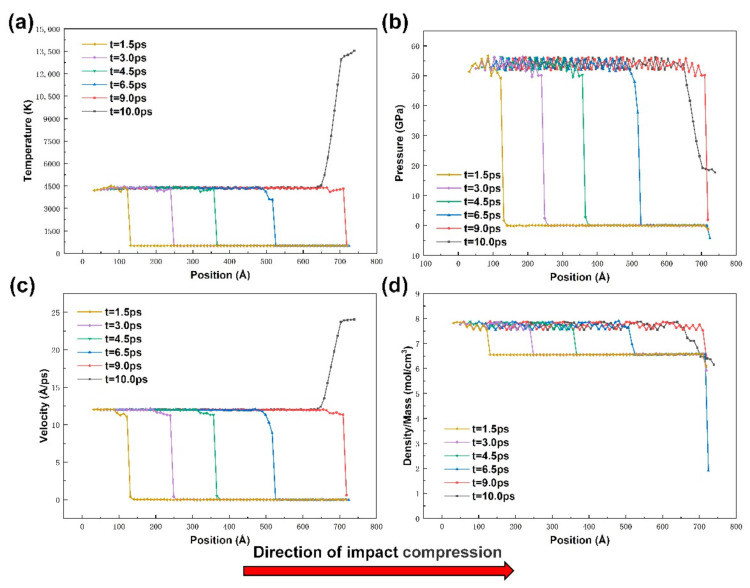
Variation curve of average value of each atom in all physical quantities during impact compression of non-porous samples: (**a**) temperature; (**b**) pressure; (**c**) velocity; (**d**) ratio of density to mass.

During the shock wave loading process, the material is compressed at an ultra-high rate, leading to changes in a series of physical quantities, such as temperature, stress, velocity, density-to-volume ratio, etc. In this paper, the sample model was divided into 3 a equal parts along the z-axis direction (a is the lattice constant), and the atomic parameters in each sample were averaged, as shown in Figure 7, for the distribution of particle temperature, stress, velocity, and density-to-mass ratio along the sample z-axis direction at *t* = 1.5 ps, *t* = 3.0 ps, *t* = 4.5 ps, *t* = 6.5 ps, *t* = 9.0 ps, *t* = 10.0 ps, etc., for the non-porous sample curves. It can be seen from the figure that with the passage of time, the shock wave propagates uniformly from the left end to the right, and the wave velocity Us is approximately 78.3 Å/ps. The temperature, stress, atomic velocity, and material density in the post-shock wave region rises significantly and remains uniform, and there is an obvious step at the leading edge of the propagation curve at each moment, and this step region corresponds to the wave front surface, and the physical quantities such as temperature and velocity in the wave front surface region are slightly lower than those in the post-wave region. The propagation curve and microstructure correspondence will be introduced in the next section for detailed comparison. At *t* = 9.0 ps, the shock wave front is already close to the surface of sample B. At *t* = 10.0 ps, the atomic velocity and temperature near the surface of B increase sharply, while the impact stress and density-to-mass ratio decrease significantly, and the density-to-mass ratio even drops below the initial value, indicating that the shock wave has reached surface B of the sample and produced reverse stretching, which is consistent with the energy propagation results shown in Figure 5.

**Figure 8:**
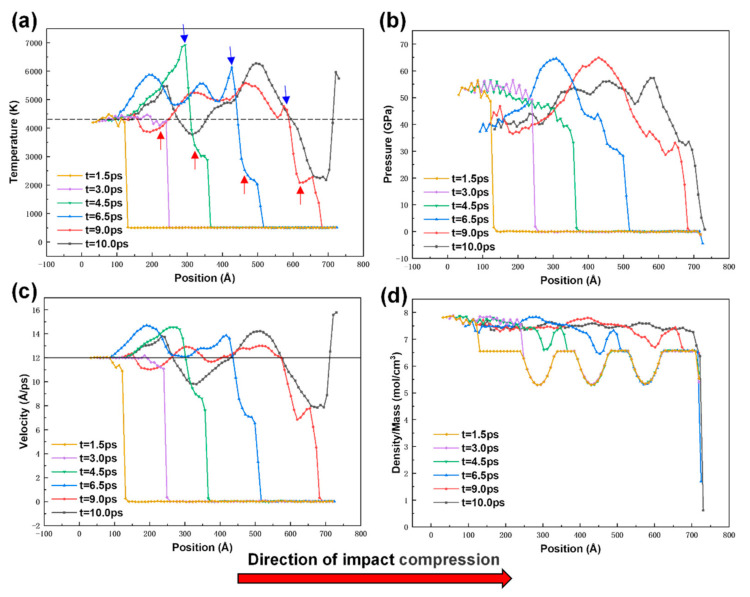
Variation curve of average value of atoms in all physical quantities during impact compression of porous samples: (**a**) temperature; (**b**) pressure; (**c**) velocity; (**d**) ratio of density to mass (the dotted line shows the average temperature and the solid line shows the average speed of the impact compression process of the non-porous sample).

Figure 8 shows the distribution curves of particle temperature, stress, velocity, and density-to-mass ratio along the z-axis of the sample at *t* = 1.5 ps, *t* = 3.0 ps, *t* = 4.5 ps, *t* = 6.5 ps, *t* = 9.0 ps, and *t* = 10.0 ps for the porous sample. Figure 8a,c at *t* = 1.5 ps and *t* = 3 ps show that the shock wave has not yet touched the hole at this time, and the waveform curves of shock wave propagation are the same as those at the corresponding moments in Figure 7. Analysis of the waveforms corresponding to the moments *t* = 4.5 ps, *t* = 6.5 ps, and *t* = 9.0 ps shows that when the shock wave encounters the hole, the temperature and the internal atomic velocity in the hole region rise sharply, and there are several more wave peaks compared to the velocity profile of the non-hole sample; the foremost wave peak at 6.5 ps is lower than the foremost wave peak at 4.5 ps, and the foremost wave peak at 9.0 ps is lower than that at 6.5 ps, as shown by the blue arrow in the figure. From the above results, it can also be predicted that if another hole is present later, the peak of the foremost wave will be lower than the mean value of the sample without the hole (dashed line in Figure 8a). In addition, as shown by the red arrows in the figure, the height of the shock wave front at 4.5 ps is lower than the height corresponding to 3.0 ps, and the height of the shock wave front at 6.5 ps is lower than the height corresponding to 4.5 ps. The height of the shock wave front gradually decreases as the number of holes through which the wave propagates increases. These two significant change patterns thus indicate that the presence of holes reduces the wave propagation energy, thus showing a buffering effect on the shock waves. Comparing Figure 7a and Figure 8a, the leading edge of the shock wave front of the sample without holes propagates to 720 Å at *t* = 9.0 ps, while the leading edge of the shock wave front of the porous sample propagates to 680 Å, indicating that the presence of holes reduces the propagation speed of the shock wave. At this time, the propagation velocity Us of the shock wave in the porous sample is about 66.7 Å/ps, while the propagation velocity of the shock wave in the non-porous sample is still about 78.3 Å/ps. The analysis of Figure 8d at *t* = 10.0 ps shows that the mass/density ratio tends to be uniform throughout the sample, indicating that the holes have been filled with atoms, which is consistent with the results of the atomic structure evolution demonstrated in Figure 3b.

### 3.5. Influence of Voids on the Shock Wave Front

**Figure 9:**
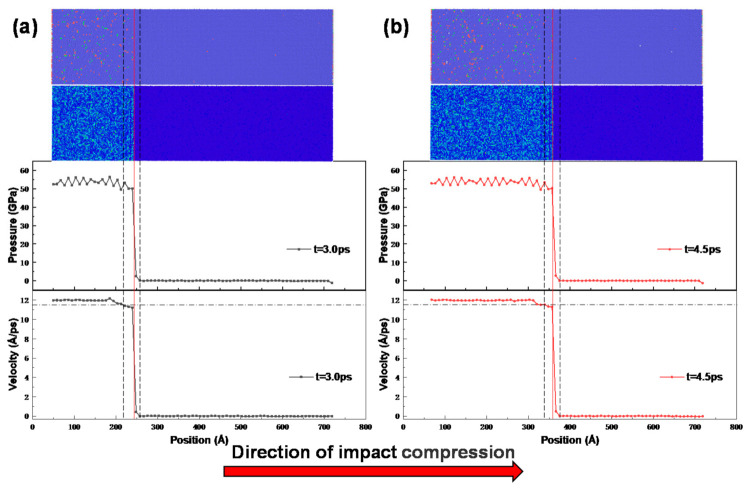
Cross-sectional view of atomic structure, energy propagation, stress, and particle velocity of non-porous NiTi samples after impact compression: (**a**) 3.0 ps; (**b**) 4.5 ps (the black chain line marks the position of 95% maximum velocity, the black dotted line marks the position of the shock wave front, and the red line indicates the elastic–plastic transition point).

A shock wave front is a stress or free surface velocity history profile along the impact loading direction that can macroscopically reflect the mechanical response of a metallic material during the impact compression phase [37]. The shock wave front is an abrupt surface with a certain transition zone. Figure 9 shows the potential energy, stress profile, and particle velocity profile of the non-porous NiTi sample at 3.0 and 4.5 ps after 1.2 km/s shock compression. The width of the shock wave front is defined [38] as the distance between the particle velocity, going up from 0% to 95%, as shown by the black dashed lines in Figure 9a,b. From Figure 9a,b, it can be seen that the wave front widths of the non-porous NiTi samples at 3.0 and 4.5 ps are basically equal, which is mainly due to the shorter time interval and less energy dissipated due to plastic deformation or phase change, which makes the width of the shock wave front not increase significantly.

**Figure 10:**
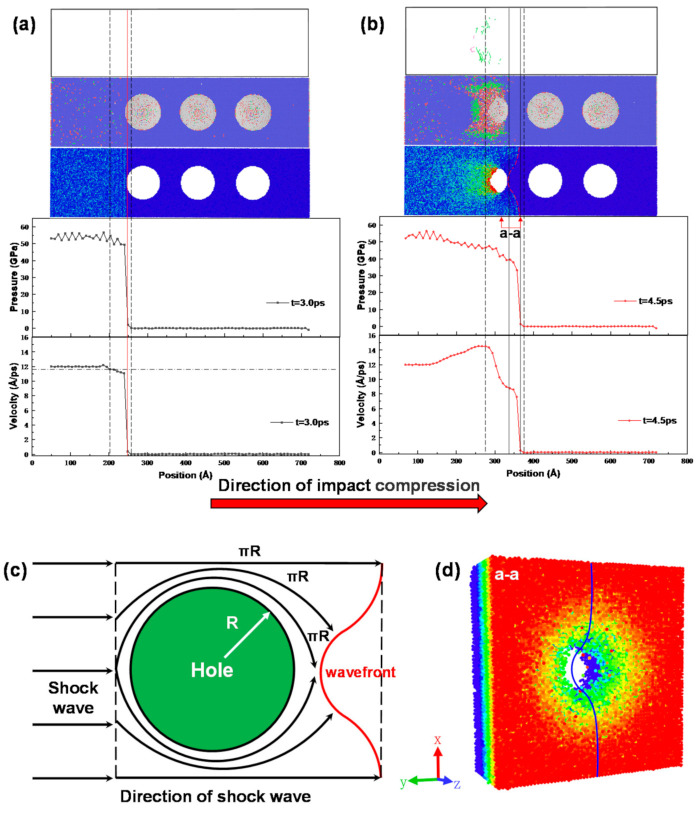
Cross-sectional view of atomic structure, energy propagation, stress, and particle velocity of porous NiTi samples after impact compression: (**a**) 3.0 ps; (**b**) 4.5 ps (the black chain line indicates the position of 95% maximum velocity, the black dotted line indicates the position of the shock wave front, the red solid line indicates the elastic–plastic transition point, the red dotted line indicates the shape of the front edge of the porous sample wave front, and the black solid line indicates the range of the front edge when the multi-space sample shock wave passes through the hole); (**c**) schematic diagram of the propagation path of the shock wave; (**d**) three-dimensional view of the wave proof shape at position a-a in (**b**) (the blue solid line in (**d**) is a meridian of the wave front shape).

Figure 10 shows the DXA dislocation identification results, potential energy distribution, stress profile, and particle velocity profile of the porous NiTi samples at 3.0 and 4.5 ps after 1.2 km/s impact compression. It is obvious from Figure 10a,b that the width of the shock wave front of the porous sample at 4.5 ps is larger than its width at 3.0 ps. The shock wave front in Figure 9 and Figure 10 reflect the mechanical response of the non-porous and porous samples during impact compression, and the process of the impact phase change can be seen. Firstly, the material is subjected to elastic wave action during compression (right side of the red solid line) and the average particle velocity and average stress of both the non-porous and porous samples are basically linearly correlated, and the atomic potential energy corresponding to the elastic wave phase is low and does not change significantly from the initial moment; then, the stress encounters the elastic–plastic inflection point and the material starts to be subjected to plastic wave action (left side of the red solid line), followed by phase transitions of different degrees, the degree of which is correlated with the presence of pores. Comparing Figure 9 and Figure 10, it is easy to conclude that the presence of the hole makes the shock wave front broaden in the same short time interval. According to Figure 4 and the conclusions in the above subsection, when the shock wave encounters a hole, the porous NiTi sample undergoes phase transformation and plastic deformation, as shown in the DXA dislocation identification results in Figure 10b, and the presence of plastic deformation leads to a sharp energy dissipation and is accompanied by the generation of dislocations, thus causing the spreading of the shock wave front. In addition, the shape of the shock wave front is concave and circular (red dashed line in Figure 10b) when the shock wave of the multi-null sample passes through the hole, and the bottom of the arc corresponds to the moment of the velocity drop and plateau in Figure 10b. It is obvious that the presence of the hole causes the shock wave front to change from a flat surface to an inwardly concave surface.

Figure 10c,d shows how the shape of the wave front surface is formed when the shock wave passes through the hole. Figure 10c shows that since there is no propagation medium inside the hole, a portion of the shock wave directly opposite the hole needs to travel forward around the edge of the hole. Since the speed of the shock wave is the same, the shock wave facing the hole needs to travel more to catch up with the shock wave not passing through the hole, i.e., the shock wave facing the hole has a certain delay relative to the shock wave not passing through the hole. Therefore, the shape of the wave front surface changes from a plane to a concave surface due to the shock wave passing through the hole. The three-dimensional view of the wave front surface is shown in Figure 10d.

## 4. Conclusions

In this paper, the impact compression behavior of non-porous/porous NiTi SMA single crystals at 500 K was investigated using large-scale molecular dynamics simulations, and the following conclusions were drawn.

(1) The B2 austenite phase transforms into B19’ martensite but not completely into B19’ phase during the impact compression stage for both non-porous and porous samples. The phase transformation region is basically in the pores after impact loading, as well as the region around the pores, and produces phase-transformation-induced plastic deformation.

(2) Under the same impact loading conditions, the shock wave propagates uniformly in the non-porous sample, but when the shock wave passes through the hole region of porous material, the original hole region collapses, and a large number of defects and dislocations are formed. This means that the existence of holes breaks the orderly propagation of shock waves, which leads to the increase in dislocation density and energy loss in the subsequent propagation process.

(3) Under the action of shock waves, the width of the shock wave front increases after passing through the holes, and the wave front surface changes from flat to curved. As the number of holes increases, the peak of the foremost wave peak gradually decreases, and the propagation speed of shock waves in the porous sample decreases. After passing through the third hole, the propagation speed of the shock wave is reduced by about 11 Å/ps, and the propagation energy of the shock wave is reduced by about 27%. These results indicate that the existence of holes reduces the propagation energy and propagation speed of shock waves.

## Figures and Tables

**Table 1:** Specific parameters of NiTi shape-memory alloy molecular dynamics model.

Holes	*L*_x_/a	*L*_y_/a	*L*_Z_/a	Number of Model Atoms
Non-porous	180	180	720	1,728,000
Porous	180	180	720	1,644,384

Note: a—lattice constant, equal to 3.014 Å; *L*_x_*—*size of X axis; *L*_y_*—*size of Y axis; *L*_Z_*—*size of Z axis.

**Table 2:** Potential function parameters.

	Ni-Ni	Ti-Ti	Ni-Ti or Ti-Ni
*D* (Å)	2.49	2.95	2.607
*A* (eV)	0.104	0.153	0.3
*P*	11.198	9.253	7.9
*ξ* (eV)	1.591	1.879	2.48
*Q*	2.413	2.513	3.002
*C*_3_	27.3341	122.395	47.8513
*C*_2_	−7.54308	−34.205	−12.92362
*C*_1_	−0.26286	−1.0054	−0.572708
*C*_0_	0.13561	0.59012	0.248676

## Data Availability

Not applicable.
